# Optical Sectioning and High Resolution in Single-Slice Structured Illumination Microscopy by *Thick Slice* Blind-SIM Reconstruction

**DOI:** 10.1371/journal.pone.0132174

**Published:** 2015-07-06

**Authors:** Aurélie Jost, Elen Tolstik, Polina Feldmann, Kai Wicker, Anne Sentenac, Rainer Heintzmann

**Affiliations:** 1 Institute of Physical Chemistry, Abbe Center of Photonics, Friedrich-Schiller University Jena, Jena, Germany; 2 Leibniz-Institute of Photonic Technology, Jena, Germany; 3 Carl Zeiss AG, Corporate Research and Technology, Jena, Germany; 4 Institut Fresnel, CNRS, Aix-Marseille Université, Marseille, France; 5 King’s College London, Randall Division, London, United Kingdom; University of California, Berkeley, UNITED STATES

## Abstract

The microscope image of a thick fluorescent sample taken at a given focal plane is plagued by out-of-focus fluorescence and diffraction limited resolution. In this work, we show that a single slice of Structured Illumination Microscopy (two or three beam SIM) data can be processed to provide an image exhibiting tight sectioning and high transverse resolution. Our reconstruction algorithm is adapted from the blind-SIM technique which requires very little knowledge of the illumination patterns. It is thus able to deal with illumination distortions induced by the sample or illumination optics. We named this new algorithm *thick slice* blind-SIM because it models a three-dimensional sample even though only a single two-dimensional plane of focus was measured.

## Introduction

Research in fluorescence microscopy is increasingly directed towards 3D imaging and several techniques such as three-dimensional (3D) Structured Illumination Microscopy (SIM) now provide 3D images with high transverse and axial resolution of living biological systems [1], albeit at the expense of demanding significant experimental complexity. However, if the biological problem can be solved by acquiring only a single focal slice despite of the sample being truly three-dimensional, many experimental problems can be overcome.

In SIM, the fluorescent labeled sample is typically illuminated with a sinusoidal pattern (hereafter referred to as the illumination grating) in order to down-modulate sample frequency information that was previously inaccessible into the support of the optical transfer function [2, 3]. This principle can be used to improve the optical sectioning [4] and the transverse resolution. However, the SIM image reconstruction is very sensitive to any error on the grating position, periodicity and overall shape [5, 6]. Recent developments allowed the reconstruction of SIM images of thin samples even with distorted or unknown pattern [7], but these algorithms are incapable of dealing with samples being truly three-dimensional. This makes SIM particularly difficult to use with thick samples which are more likely to distort the excitation pattern.

Here, we present a reconstruction algorithm, hereafter named *thick slice* blind-SIM, capable of processing SIM *single slice* data acquired in *thick* samples. Our approach is inspired by the rencently developed deconvolution-based reconstruction method called blind-SIM in which the illumination pattern is reconstructed along with the object [7, 8]. Since blind-SIM does not require the knowledge of the illumination pattern, it is more robust to experimental imprecision and possible sample-induced distortion than classical SIM reconstruction approaches, while maintaining high resolution and tight optical sectioning abilities. Up to now, blind-SIM has been developed in a strict two-dimensional framework only compatible with very thin samples. Any out-of-focus contribution caused the algorithm to fail. The main idea of *thick slice* blind-SIM is to process the 2D data with an alternate 3D deconvolution over the sample and illuminations but accounting for incomplete measured data, thus having the ability to reject the out-of-focus blur.

## Methods

**Principle of blind-SIM**. The imaging process in a SIM microscope can be described by

$$I_{det,l}=(\rho \cdot I_{illu,l})⊗h+𝓝\;,$$ (1)

where *I*_*det*,*l*_ is the *l* ^*th*^ detected image, *ρ* is the sample *e.g*. the distribution of fluorophores, ⊗ stands for the convolution operator, *I*_*illu*,*l*_ is the *l* ^*th*^ illumination grating, *h* is the point spread function (PSF) and 𝓝 accounts for the noise. *ρ* describes the biological reality, whereas the variable denoted $\hat{\rho }$ in Eq 2 below is an estimate of this reality. The blind-SIM algorithm described below reconstructs both the sample information $\hat{\rho }$ and the family of gratings ${\left\{{\hat{I}}_{illu,l}\right\}}_{l=1..L}$. Here, *L* = 9 since we assume 3 lateral shifts of the grating in each of the 3 directions. The reconstruction is done by minimizing the functional

$$F\left(\hat{\rho },{\hat{I}}_{illu,l}\right)=\sum_{l=1}^{L}{|[\hat{\rho }\cdot {\hat{I}}_{illu,l}]⊗h-I_{det,l}|}^{2}.$$ (2)

More precisely, the algorithm estimates values of the object $\hat{\rho }$ and the gratings $\left\{{\hat{I}}_{illu,l}\right\}$, projects these estimates according to the model (Eq 1) to calculate the predicted image, and then evaluates the quality of this prediction by calculating the least square error to the measured image *I*_*det*,*l*_. Contrary to the blind-SIM algorithm presented in [7], our algorithm is based on an update scheme that alternates between object and illumination updates, $\hat{\rho }$ and $\left\{{\hat{I}}_{illu,l}\right\}$. Similar schemes are common [9, 10]. This choice was made to keep the mathematics and the code simple and fast. Each iteration *i* contains an object estimation sub-iteration, in which $\left\{{\hat{I}}_{illu,l}\right\}$ is fixed and equal to its most recent estimate. $\hat{\rho }$ is thus updated and subsequently fixed for the illumination estimation sub-iterations, in which $\left\{{\hat{I}}_{illu,l}\right\}$ is optimized. The object is estimated for *m* iterations and the illumination function is estimated for *n* iterations. It should be noted that the optimizer might have not yet reached a minimum within these *m* or *n* iterations. This procedure is repeated for *i* = 1..*N* cycles.

1. Initial values: ${\hat{\rho }}_{0}$ and ${\left\{{\hat{I}}_{illu,l}\right\}}_{0}$ homogeneous
2. Cycle *i*—object *m* estimation steps by approaching the zero by using the gradient of F: $\frac{\partial F\left(\hat{\rho },\{{\hat{I}}_{illu,l}\}\right)}{\partial \hat{\rho }}$ for $\{{\hat{I}}_{illu,l}\}={\left\{{\hat{I}}_{illu,l}\right\}}_{i-1}$ fixed (*m* iterations)
3. Cycle *i*—grating *n* estimation steps by approaching the zero by using the gradient of F: $\frac{\partial F\left(\hat{\rho },\{{\hat{I}}_{illu,l}\}\right)}{\partial {\hat{I}}_{illu,l}}$ for $\hat{\rho }={\hat{\rho }}_{i}$ from previous step and fixed (*n* iterations)
4. End of cycle *i*: estimated values of $\hat{\rho }$ and $\left\{{\hat{I}}_{illu,l}\right\}$ updated.
5. Go to step 2 and repeat for cycle *i* + 1 until *i* = *N*

In practice, we found that *m* = 5 for the first cycle *i* = 1, *m* = 25 henceforth and *n* = 5 yield good results. The toolbox was implemented in MATLAB (R2012a, Mathworks, Natik, MA, USA) and the gradient-based optimization process uses the *minFunc* function developed by Mark Schmidt and freely downloadable from [11]. The descent direction is computed using LBFGS, which is a quasi-Newton limited memory BFGS search direction method [12]. The line search strategy, which determines the step length, is based on the strong Wolfe condition (with a cubic interpolation strategy). The initial step size is the minimum between 1 and twice the previous step length. It should be noted that a recently published blind reconstruction scheme [10] termed “patterned-illuminated Fourier Ptychography” may at first sight seem different, but is in fact identical if updating is performed after every single pattern comparison step by steepest descent with a step length of one. For the special case of assuming Gaussian noise of constant variance (as used in this manuscript), we can also compute the residual in Fourier space, thus avoiding two Fourier transformations per iteration cycle.

**The novelty of our** ***thick slice*** **implementation** is that, even though 2D data were acquired, a 3D deconvolution is performed. The algorithm calculates with a full three-dimensional volume of *P* focal slices, but in the comparison step between the 3D data predicted from the 3D guess and the 2D measured data, only the central slice is computed and all other comparison values are simply set to zero at this step. The whole stack is then propagated back from measurement space into object space as typically done for gradient-based maximum-likelihood methods [13]. The algorithm minimizes Eq (2), with *h* being the 3D PSF. We therefore perform a three dimensional deconvolution and the object estimates are three dimensional despite the data being two-dimensional in nature. Due to the axial extent of the PSF, information—the out-of-focus contribution present in the focal slice—will propagate to and accumulate in the extra planes during the deconvolution process and the initially empty planes in 3D sample estimate are filled. The error is calculated as described above in the middle slice only, without other planes contributing. Thus, the algorithm is free to place any information into these extra planes with the goal to decrease the error in the middle plane. The user can choose the number of reconstructed planes *P* according to the sampling of the discretized PSF, denoted *scZ* in Table 1. *P* should be large enough to enable out-of-focus contribution to be efficiently rejected. However, increasing it further unnecessarily increases the computational time.

**Table 1:** Reconstruction parameters.

Fig#	P	scZ [nm]	*σ*	N	Reg. type	*λ*
Fig 1b	1	N.A.	14	40	N.A.	N.A.
Fig 1c	1	N.A.	N.A.	100	GS	1⋅10^−2^
Fig 2b	1	N.A.	N.A.	100	GS	1⋅10^−2^
Fig 2c	9	200	N.A.	100	GS	1⋅10^−3^
Fig 2e	1	N.A.	5	50	GS	7⋅10^−1^
Fig 2f	9	200	3	100	GR	5
Fig 2i	9	200	13	100	GR	20
Fig 3a	1	N.A.	N.A.	100	GS	1⋅10^−3^
Fig 3b	1	N.A.	6	50	GR	2⋅10^3^
Fig 3c	30	91	N.A.	100	GS	1⋅10^−5^
Fig 3d	13	91	6	100	GR	20

For each blind-SIM reconstruction, a number of parameters can be tuned. In this table, we summarize the chosen parameters for the presented results. The number of planes corresponds to the number of planes in a double-sided PSF. Using a half-sided PSF, this value is divided by two as the PSF contains each plane only once.

To increase the computational efficiency, we use only a half-sided PSF to make use of the z-symmetry of unaberrated PSFs. Indeed, using a “normal” PSF which is double-sided leads to identical information content on both sides of the middle plane. In practice, we simply neglect the lower half of the PSF and replace this part of the PSF with the cyclic extension of the upper part of the PSF. In other words, we use only the upper part of a PSF of double size but place the focus cyclically at the middle plane. We can thus prevent the redundancy on both sides of the middle plane. This gives an identical result as a “normal” double-sided PSF, but saves a factor of almost two in both computational time and memory. Finally, another symmetry that can be exploited is the real-valuedness of the object and measured data: a factor of two in speed is gained by using the half complex space Fourier Transform (RFT). A GPU (NVIDIA GeForce GTX 690) was used to speed up the processing (25 min vs. 330 min without GPU). Our computer has 8 processors (Intel Core (TM) i7—3770 CPU @3.40 GHz) and 32 GB memory.

**Further regularization and constraints** were introduced to make the algorithm more powerful. First, we apply a condition stating that the sum of each triplet of gratings (for each direction) is uniform. Thus, we estimate only two (resp. *J* − 1 if *J* is the number of illumination patterns per direction) out of every three (resp. *J*) unknown illumination patterns and obtain the remaining ones using this sum condition. This reduces the number of unknowns of the system [7]. This condition should be fulfilled experimentally for a typical situation where relative pattern shifts are well controlled, but aberrations still being present, deforming the images of those patterns. The phase steps applied to a sinusoidal grating should be equal. In the appendix S1 Appendix, we present a detailed discussion concerning aberrations and show that the sum condition is still fulfilled even if the interfering wavefronts are aberrated.

Second, neither the reconstructed object nor the illumination should contain negative values since we are in the framework of fluorescence imaging. The non-negativity of the solution could be applied in the form of a penalty term [13], but we found that intrinsic positivity as in [7] for the sample estimate yielded nicer results.

We therefore wrote $\hat{\rho }$ as the square of an auxiliary function *ξ*.

$$\hat{\rho }=\xi^{2},$$ (3)

As commonly done for this class of problems which are typically ill posed, we introduced a regularization term. For smoothness constraints, Good’s Roughness (GR), gradient square (GS) or the hyperbolic Total Variation (hTV) regularization functions were applied [14, 15]. The regularization term is added to the error with a weight *λ* in the object estimation step. Practically, we tuned the type and *λ* of the regularization for each sample. The choice has to be made as a compromise between resolution and smoothness of the result. No smoothness constraint was applied to the illumination function. In [7], no regularization term was applied, but the iterations were stopped at a user-defined number to prevent over-deconvolution artefacts.

Furthermore, *partial* knowledge of the grating, if available, can be used by using an illumination mask in Fourier space. A round kernel of radius *σ* is placed at the expected positions of the peaks of the Fourier Transform (FT) of the grating. In the illumination iteration step, the algorithm searches for solutions exclusively inside of this Fourier mask. This constraint makes the algorithm more robust, *e.g*. the high frequencies are reconstructed with better contrast, while accounting for potential experimental imprecisions. For instance, the grating period may be slightly different than in theory, or the fringes may be distorted, and yet the grating will still be reconstructed [16].

In practice, the proposed method requires a priori known input parameters (e.g. the approximate grating constant) and tunable parameters such as the choice of regularization method and its weight factor. This has the disadvantage of introducing subjectivity, but also constraints the solution to a more reasonable result, which is also of practical importance. A possibility to avoid the need for regularization is the use of bootstrapping schemes [17], which come, however, at the cost of significantly increased computation time. In Table 1, we state the reconstruction parameters used for each figure. We were so far unable to provide a proof of convexity of our alternating approach. However, we observed a fast convergence to useful results in all cases with the parameters set as given in Table 1.

## Results

### Proof-of-principle

**Fig 1:**
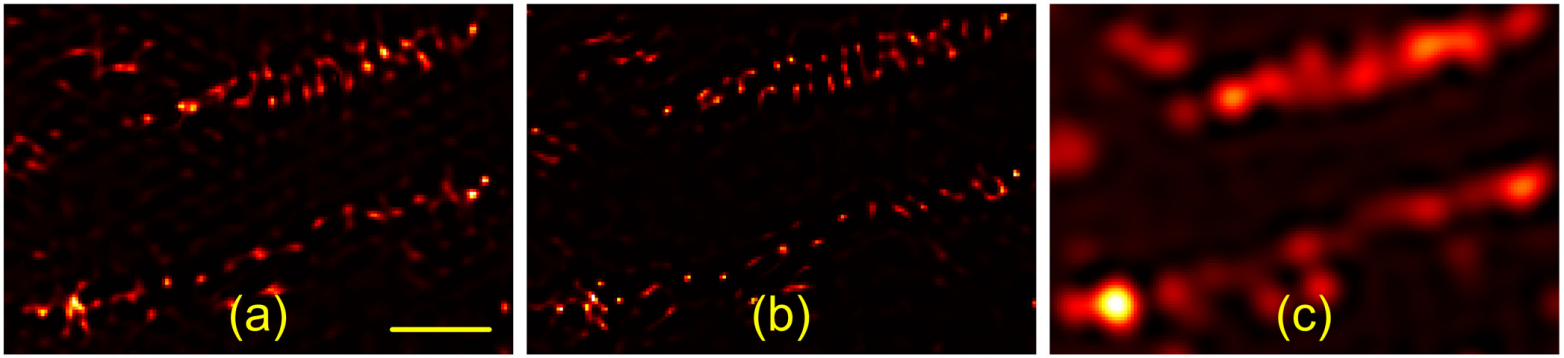
Comparison of the different existing 2D blind-SIM algorithms on a paxillin sample illuminated with a distorted grating. a) 2D blind-SIM using simultaneous estimation of the object and illumination functions [16]. b) 2D blind-SIM using the proposed method with sequential estimation. For improved comparability, a conjugate gradient scheme was used here. c) 2D WF deconvolution. The scale bar is 1 *μ*m.

First, we demonstrate that the *alternate* estimation method gives quite comparable results than the previously published blind-SIM algorithm [16]. In Fig 1, we compare both approaches on an ultra-thin paxillin labelled cell illuminated with a distorted grating. We applied the previously published [16] 2D blind-SIM approach (Fig 1a). As seen from Fig 1, both our deconvolution (Fig 1b) and the previously published result (Fig 1a) [[16]] show a roughly similar information content, especially when compared to the deconvolved wide-field image (Fig 1c). There are visible differences especially in the amount of thread-like appearances connecting the dot-like patterns. As we lack the underlying ground-truth information, we are unable to decide which of the reconstruction result is closer to reality. Next to the joint optimization scheme, a further difference to our implementation is that, in [16], the authors did not use any regularization, but instead tuned by hand the number of iterations to stop the minimization before noise start to be amplified. Our maximum a posteriori likelihood [MAP] approach is a bit different.

### Simulations

**Fig 2:**
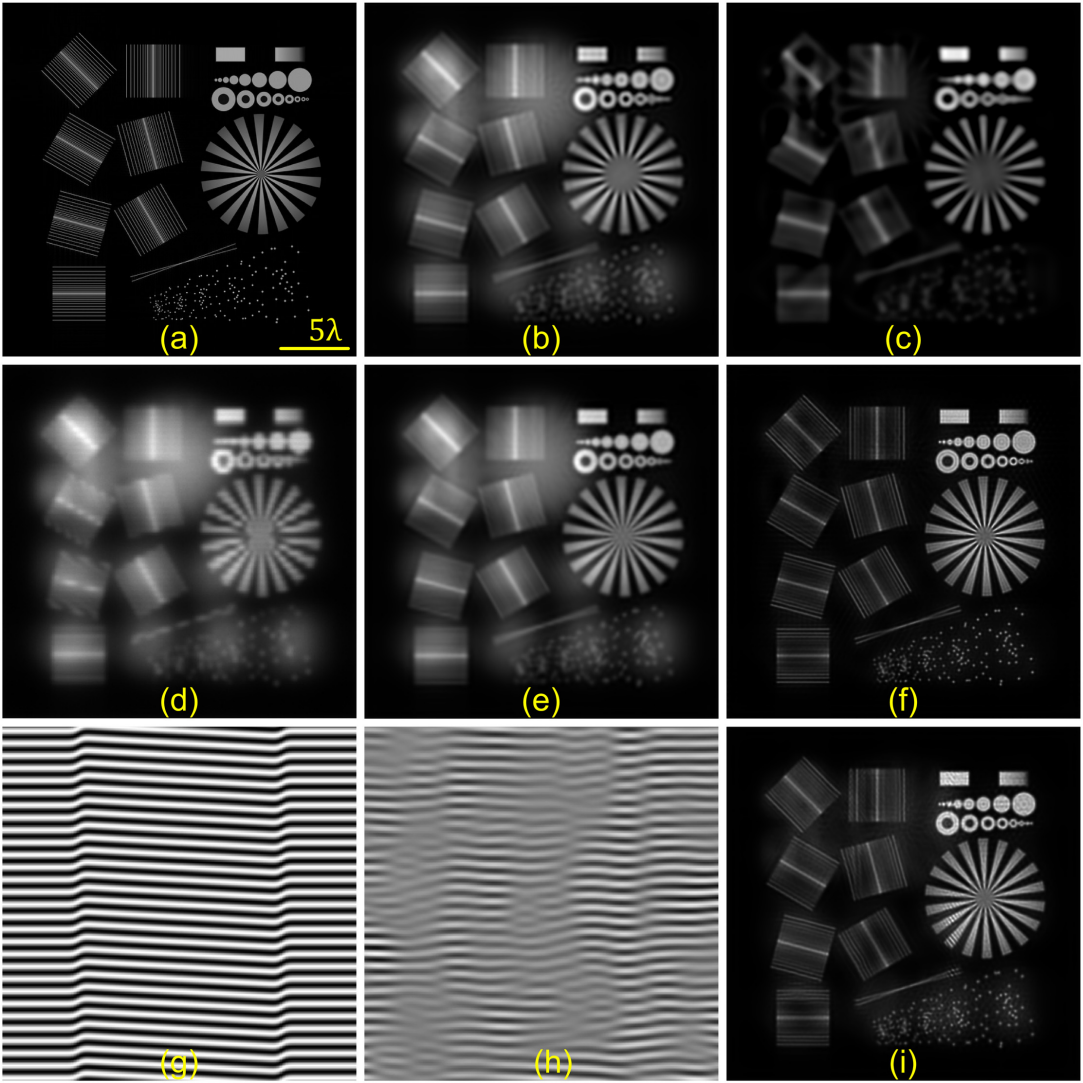
Results of 3D simulation with ***thick slice*** reconstruction. a) Resolution test target placed in the focal plane of our simulated sample. 800nm out-of-focus was a *π*/2-rotated version of the same structure. b) 2D WF deconvolution. c) Focal slice of 3D WF deconvolution of the entire WF image stack. d)One of the 9 simulated SIM images. Here we simulate a two-beam SIM. e) 2D blind reconstruction of (d) containing out-of-focus light. f) *Thick slice* blind-SIM result, showing optical sectioning and high resolution. g-i) Simulation as in a-f) but with a distorted illumination pattern as depicted in g) (zoom). h)Reconstructed illumination function. i) *Thick slice* blind-SIM reconstruction of the object described in (a) but illuminated with the distorted pattern(g). Scale bar: 2.5 *μ*m.

In order to test our *thick slice* blind-SIM algorithm, we used a simulated object consisting of several practical resolution test targets (Fig 2a) [5]. We introduced out-of-focus information by placing a *π*/2-rotated version of the same object 800nm displaced along the axial direction. Two-beam illumination was simulated by multiplying this object with a grating of 214nm period, that was positioned three times by 2*π*/3 in the 0°, 60° and 120° orientations in the plane. Poisson noise was introduced in the data so that the expected value of the brightest pixel in the sum of all images corresponds to a count of 4 ⋅ 10^5^ photons. The data were then convolved by a 3D PSF, simulating imaging through a microscope objective of numerical aperture (NA) 1.3 at an emission wavelength of 500nm. The total object was 512 × 512 × 9 voxels, with a square voxel size of 25 × 25 × 200nm^3^.

Out of this 3D stack, only the focal plane data was selected. One of the 9 images with visible out-of-focus contributions is depicted in Fig 2d. The wide-field (WF) images for comparison were obtained by summing all individual raw SIM images. First, the 2D blind-SIM algorithm was applied to the single-slice data (Fig 2e). We observe that the out-of-focus blur is still present. We then applied the *thick slice* blind-SIM algorithm with the reconstruction parameters as presented in Table 1. The middle plane of the *thick slice* result (Fig 2f) contains no out-of-focus light. The comparison of Fig 2e with the 2D WF deconvolution (Fig 2b) and of Fig 2f with the 3D WF deconvolution, here using the entire simulated stack data rather than just the single slice data (Fig 2c), demonstrates the resolution enhancement. We also studied the case where the illumination was distorted as shown in Fig 2g. *Thick slice* blind-SIM reconstructed an object (Fig 2i) which is, except for a slight loss in the sectioning ability, still quite similar to that obtained in the non-distorted case (Fig 2f), as well as the illumination function (Fig 2h). By comparing the simulated illuminated pattern (Fig 2g) and its reconstruction (Fig 2h), one can observe three things: first, blind-SIM can only reconstruct the illumination function well for the regions in the field where there is information, i.e. where the sample emits photons. In such a simulated sample, there are dark regions where the illumination function cannot be retrieved reliably. Second, the reconstructed frequency is correct, which means that the final resolution is optimal. Not only the frequency but also the phase distortions should be retrieved for a successful and correct reconstruction. In Fig 2h, we see that the applied phase jump, particularly on the right side of the image, was correctly recovered.

In addition to this resolution slide simulation, we tested several other simulated objects—including hollow spheres—which confirmed the optical sectioning ability of *thick slice* blind-SIM. For instance, if we place a single point out-of-focus and no information in the focal place, we manage to not only completely reject its light contribution but also to reconstruct it down to a single point in an out-of-focus plane.

### Experimental Validation

The commercially available Elyra S-1 from Carl Zeiss was used to produce the experimental data whose reconstructions are shown in this section. It is a 3D SIM system, *i.e*. three plane waves are interfering in sample space to produce the illumination modulation. A 3D set of data was available, which permitted us to compare with the 3D deconvolved WF image. Acquisition parameters were: NA 1.4, voxel size 79nm, distance between the acquired planes 91nm, excitation wavelength 488nm and coarse grating period 440nm. The sample was prepared by growing MCF-7 breast cancer cells on the coverslip with following fixation and staining of actin with “Alexa Fluor®488 Phalloidin”, emitting at 550nm.

**Fig 3:**
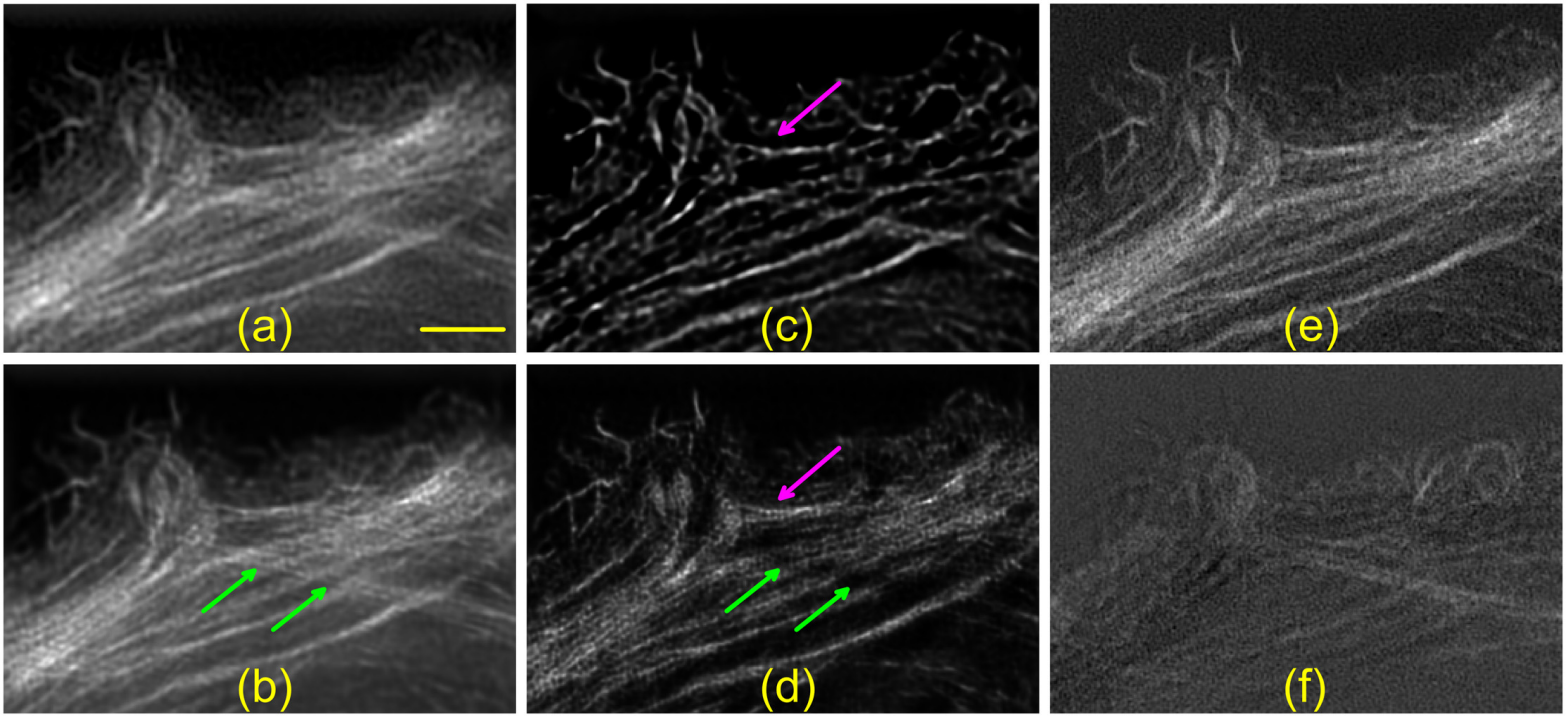
Experimental results in an MCF-7 actin-labelled cell. A 200×200 pixels region of interest was selected to keep the computational time small. a) 2D wide-field (WF) deconvolution. b) 2D blind-SIM reconstruction with higher resolution but no optical sectioning. c) 3D WF deconvolution. d) *Thick slice* blind-SIM result. The resolution is improved and the out-of-focus contribution removed. The green arrows indicate a pair of filaments that is removed because it originally stems from another plane. The processing time was 25 min with GPU vs. 330 min without GPU. e) and f) are the reconstructions of the original 3D data with the ZEN software (version 2010D). e) Plane that was selected. f) Two slices under slice e), i.e. 182 nm away. The sample was prepared by Michael Reuter and data acquired by Elen Tolstik on a commercial ELYRA-S.1 SIM microscope (Carl Zeiss Microimaging, Jena, Germany). Scale bar: 2 *μ*m.

We selected a region of interest in a given plane and performed 2D and *thick slice* blind-SIM reconstructions (resp. Fig 3b and 3d). The reconstruction parameters can be found in Table 1. In addition, we perform 2D and 3D WF deconvolutions (respectively Fig 3a and 3c).

Comparing the blind-SIM results with the WF deconvolutions demonstrates the resolution enhancement due to the structured illumination. The magenta arrows indicate two filaments that appear to be merged in the WF images but are separated in the blind-SIM results. 2D blind-SIM cannot account for out-of-focus fluorescence, leading to residual patterning in the background of Fig 3b. We observe another striking difference between 2D and *thick slice* blind-SIM, indicated by the green arrows. In Fig 3d, the indicated pair of filaments is removed, while being present in Fig 3b. It seems that these fibres did not preside entirely in the selected plane. The part which is close to the left arrow is located about 200nm out-of-focus. We could verify this information thanks to the 3D reconstruction using the entire 3D datastack calculated on the commercial instrument. In these images, the filaments are also not visible in the focal plane (Fig 3e) but in focus in another plane located 182nm away (Fig 3f).

In this case, the blind-SIM algorithm did not significantly improve the quality of the reconstructions as compared to the commercial software because the illumination was not distorted. We use this data as proof-of-principle to demonstrate that *thick slice* blind-SIM has the expected resolution enhancement and optical sectioning by processing only a single slice.

## Conclusion

We presented a state of the art implementation of blind-SIM that is able to process two-dimensional SIM data of a thick sample requiring only a partial knowledge of the illuminations. We showed on simulated and measured biological objects that *thick slice* blind-SIM provides images with an optical sectioning and lateral resolution enhancement similar to that of a 3D SIM system requiring only one single-slice acquisition.

In the future, we plan to modify the blind-SIM algorithm so that it can deal with 3D SIM serial slices in a unified model. This will be done with the help of multiple PSFs, one for each separated order. Furthermore, we plan to implement phase-only constraints and phase-only aberration recovery strategies.

## Supporting Information

S1 Appendix The appendix presents a discussion concerning aberrations based on some mathematical description, leading to the proof that the sum condition is fulfilled also in the case where the interfering wavefronts are aberrated. (PDF)

S1 Raw data This folder contains all raw data underlying the manuscript. (ZIP)
