# Supplementary material for: Optical Sectioning and High Resolution in Single-Slice Structured Illumination Microscopy by Thick Slice Blind-SIM Reconstruction
Source: PLoS One. 2015 Jul 6;10(7):e0132174. doi: 10.1371/journal.pone.0132174 (PMC4493150; doi:10.1371/journal.pone.0132174)
Supplement: S1 Appendix — (PDF) [file pone.0132174.s001.pdf]

Appendix: Aberrations and the sum condition

In situations where we want to apply our blind SIM algorithm, we can assume that the aberrations are relatively minor and a single parallel beam would not lead to significant brightness variations or otherwise only to realtively coarse brightness changes resulting a similarly modified deconvolution result. By now interfering two or more beams, of locally always correct phase steps, we can show that the incoherent sum of all coherently interfering waves leads to equal brightness which, in the worst case again, will lead to slightly wrong reconstructed sample brightnesses. However, in situations of deep tissue imaging with fully developed speckles, this assumption does not hold, however this is not the application that blind-SIM is targeted for. In the following, we assume that we are in the single scattering regime, there are only few refractive surfaces, no fully developed speckles, which means that the intensity is relatively smooth although the phase distortions can reach several multiples of π. The aberrations foremost affect the locally present phase. This seems justified when only a few cell layers are illuminated and without large refractive index difference and little absorption. As demonstrated below, even complicated phases still average out.

We have three beams corresponding to the zero and two first amplitude orders of a diffraction grating. We index the amplitude order with the letter a. In the SIM configuration, they are plane waves in the sample space.

$$E_a(\vec{r},t) = |E_a| \cdot e^{i\left(\vec{k_a}\cdot\vec{r}-\omega t+\phi_{a,l}+\chi_a(\vec{r})\right)}, \tag{1}$$

with $a = -1, 0$ or 1 and where

$$\phi_{a,l} = a \cdot \frac{l}{L} 2\pi, \tag{2}$$

for $l = 1..L$ (L equidistant phase steps). $\phi_{a,l}$ is a constant phase term determined by the diffraction grating and which can be changed by translating the grating. The aberration term is modeled as a phase term χ_a. This function describes the deformation of the wavefront and varies smoothly with position. However, it does not depend of the order phase step l. The aberration map for the two first orders are generally different from one another, as the beams do not traverse the same part of the sample. However the phase terms are maintained (caused by the shift of a diffraction grating).

The light intensity at a position $\vec{r}$ is the interference of all electric field order contributions at this spot. In the SIM case, each intensity order is the result of the interference of two amplitude orders (except for the zero order for which phase contributions cancel out). Here we denote these two orders a and b, which can each be $-1, 0$ or 1. Their intereference can be written in the following way:

$$E_a \cdot E_b^* = |E_a||E_b| \cdot e^{i\left(\left(\vec{k_a}-\vec{k_b}\right)\cdot\vec{r}\right)} \cdot e^{i(\omega-\omega)t} \cdot e^{i(\chi_a(\vec{r})-\chi_b(\vec{r}))} \cdot e^{i(\phi_{a,l}-\phi_{b,l})}. \tag{3}$$

When we sum over all L patterns we obtain for interferences between any two different orders a and b:

$$\sum_{l=1}^{L} E_a \cdot E_b^* = |E_a||E_b| \cdot e^{i\left(\left(\vec{k_a}-\vec{k_b}\right)\cdot\vec{r}\right)} \cdot e^{i(\chi_a(\vec{r})-\chi_b(\vec{r}))} \cdot \sum_{l=1}^{L} e^{i(\phi_{a,l}-\phi_{b,l})} = 0. \tag{4}$$

Sums out to zero since the phase steps are chosen to be equidistant, for example in the case where we have three equidistant phase steps:

$$\sum_{l=1}^{L} e^{i(\phi_{a,l}-\phi_{b,l})} = \sum_{l=1}^{L} e^{i\left(a\cdot\frac{l}{L}2\pi-b\cdot\frac{l}{L}2\pi\right)} = e^{i\cdot\frac{2\pi}{3}} + e^{i\cdot\frac{4\pi}{3}} + e^{i\cdot 2\pi} = 0. \tag{5}$$

In this case taking the example of a=1, b=0 and L=3 for the sake of demonstration. Note also, that only the contributions for a=b do not cancel, which thus results in illumination being well described as constant to a good approximation.
