# Supplementary figures and images for: Optical Sectioning and High Resolution in Single-Slice Structured Illumination Microscopy by Thick Slice Blind-SIM Reconstruction
Source: PLoS One. 2015 Jul 6;10(7):e0132174. doi: 10.1371/journal.pone.0132174 (PMC4493150; doi:10.1371/journal.pone.0132174)

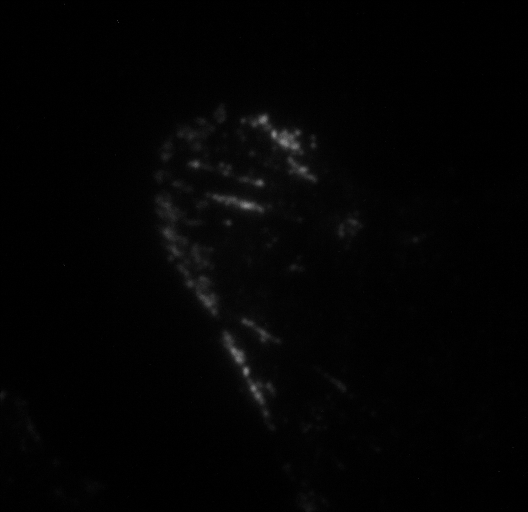

Supplement: S1 Raw data — (ZIP) [file pone.0132174.s002.zip › Final_to_upload/Fig1_paxillin_comp/pax_simD000.tif]

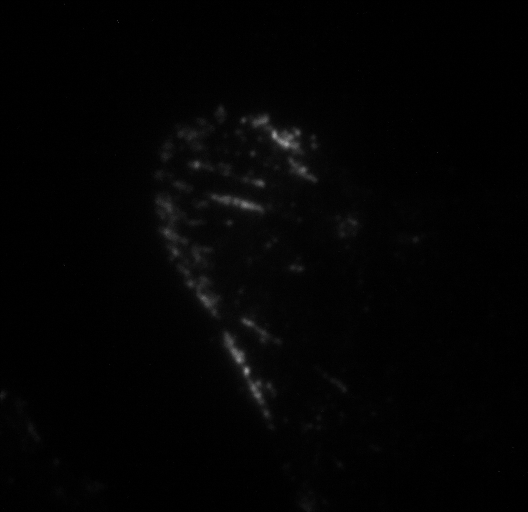

Supplement: S1 Raw data — (ZIP) [file pone.0132174.s002.zip › Final_to_upload/Fig1_paxillin_comp/pax_simD001.tif]

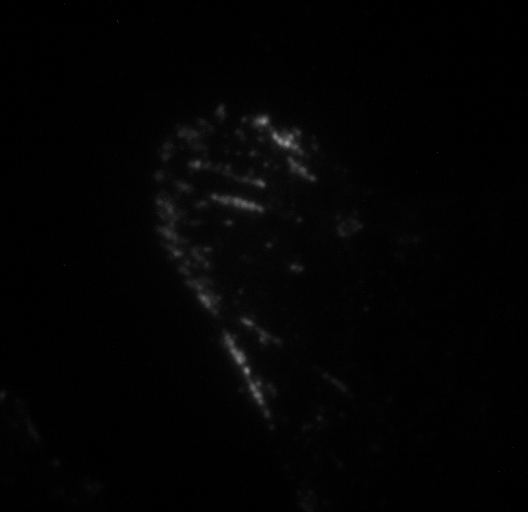

Supplement: S1 Raw data — (ZIP) [file pone.0132174.s002.zip › Final_to_upload/Fig1_paxillin_comp/pax_simD002.tif]

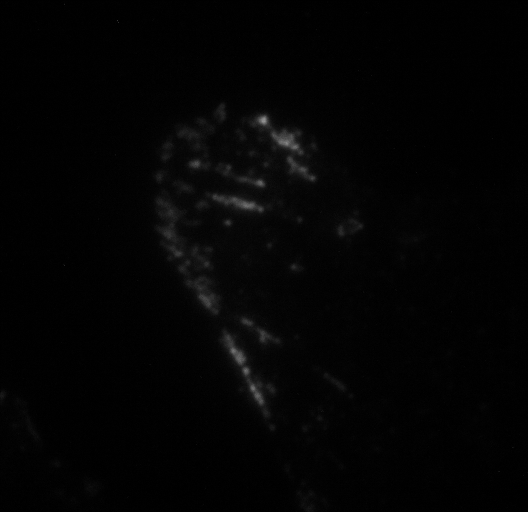

Supplement: S1 Raw data — (ZIP) [file pone.0132174.s002.zip › Final_to_upload/Fig1_paxillin_comp/pax_simD003.tif]

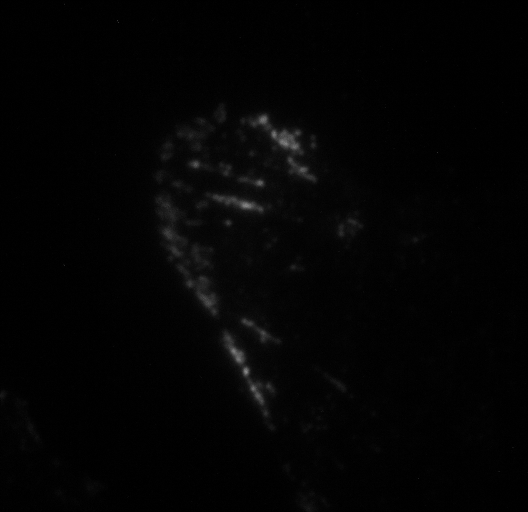

Supplement: S1 Raw data — (ZIP) [file pone.0132174.s002.zip › Final_to_upload/Fig1_paxillin_comp/pax_simD004.tif]

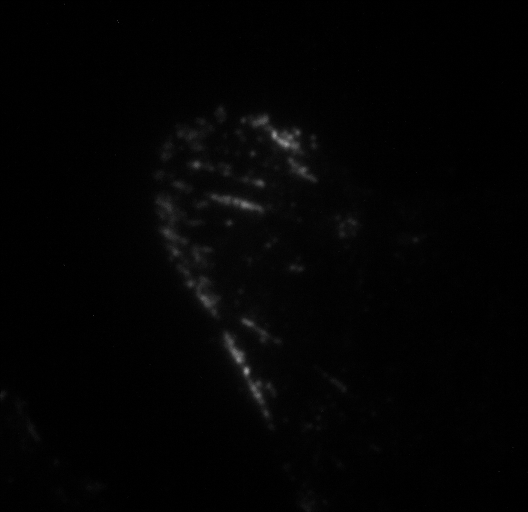

Supplement: S1 Raw data — (ZIP) [file pone.0132174.s002.zip › Final_to_upload/Fig1_paxillin_comp/pax_simD005.tif]

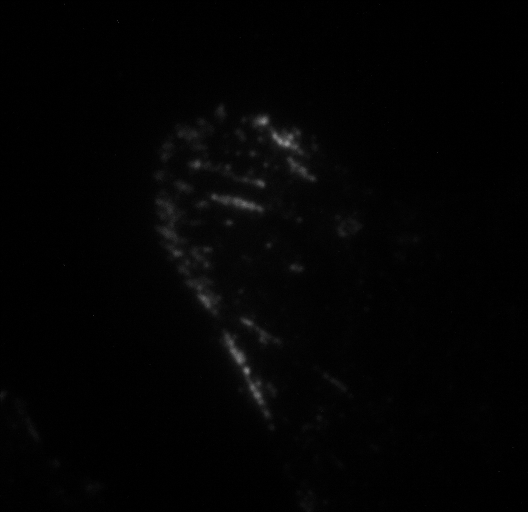

Supplement: S1 Raw data — (ZIP) [file pone.0132174.s002.zip › Final_to_upload/Fig1_paxillin_comp/pax_simD006.tif]

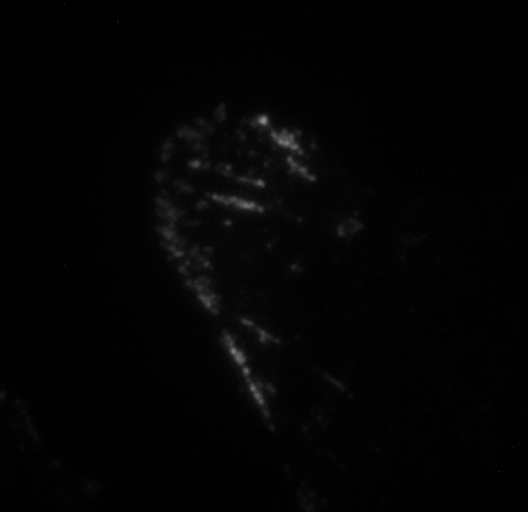

Supplement: S1 Raw data — (ZIP) [file pone.0132174.s002.zip › Final_to_upload/Fig1_paxillin_comp/pax_simD007.tif]

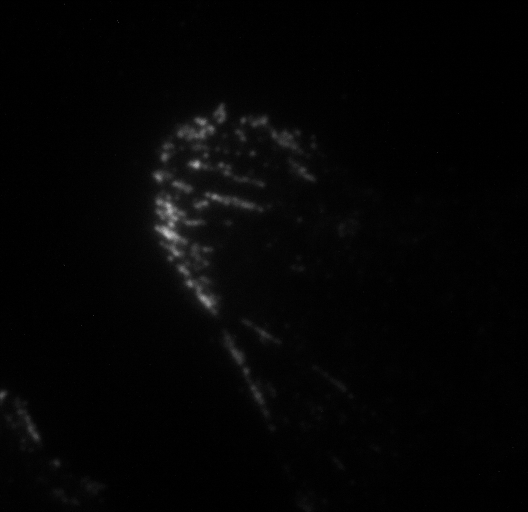

Supplement: S1 Raw data — (ZIP) [file pone.0132174.s002.zip › Final_to_upload/Fig1_paxillin_comp/pax_simD008.tif]

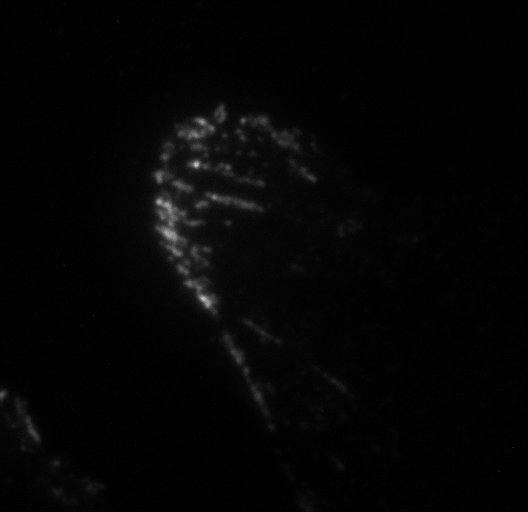

Supplement: S1 Raw data — (ZIP) [file pone.0132174.s002.zip › Final_to_upload/Fig1_paxillin_comp/pax_simD009.tif]

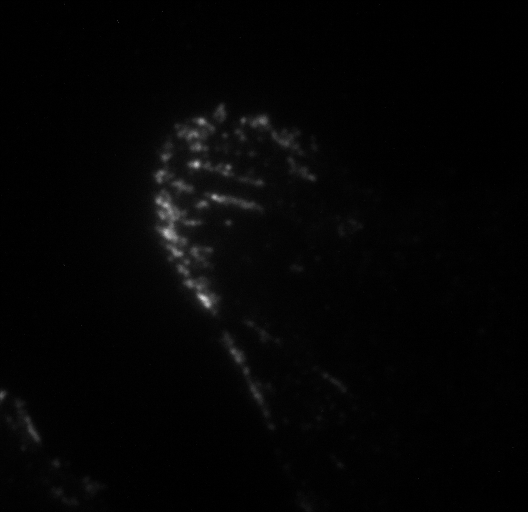

Supplement: S1 Raw data — (ZIP) [file pone.0132174.s002.zip › Final_to_upload/Fig1_paxillin_comp/pax_simD010.tif]

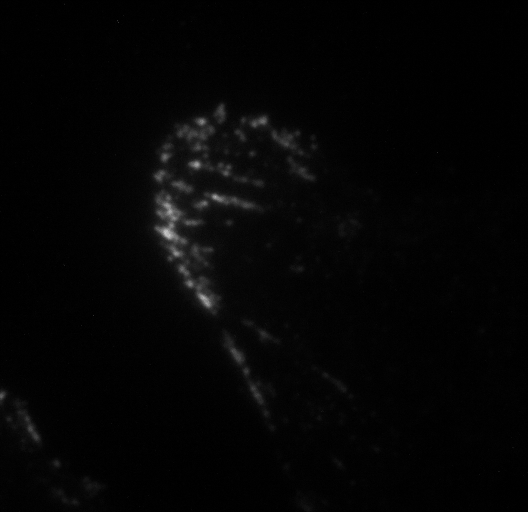

Supplement: S1 Raw data — (ZIP) [file pone.0132174.s002.zip › Final_to_upload/Fig1_paxillin_comp/pax_simD011.tif]

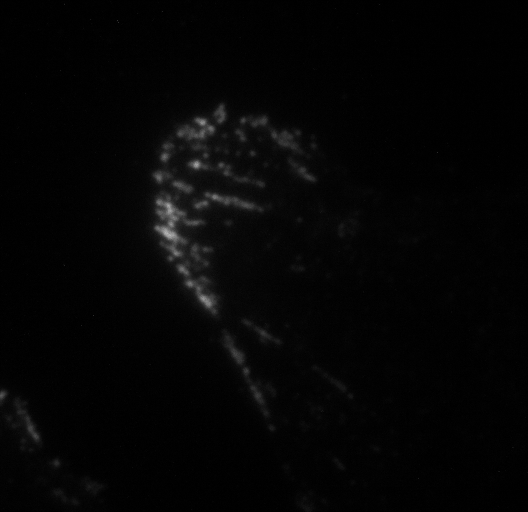

Supplement: S1 Raw data — (ZIP) [file pone.0132174.s002.zip › Final_to_upload/Fig1_paxillin_comp/pax_simD012.tif]

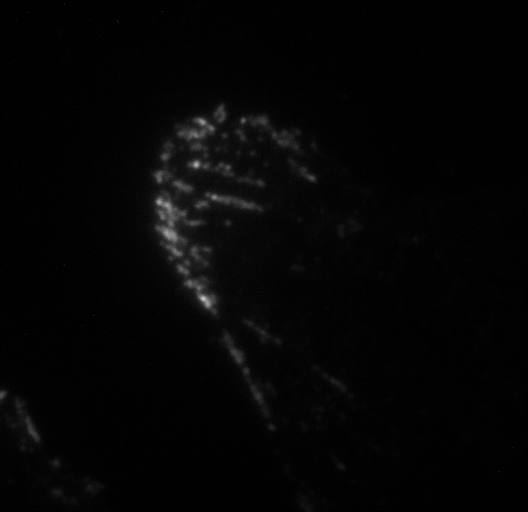

Supplement: S1 Raw data — (ZIP) [file pone.0132174.s002.zip › Final_to_upload/Fig1_paxillin_comp/pax_simD013.tif]

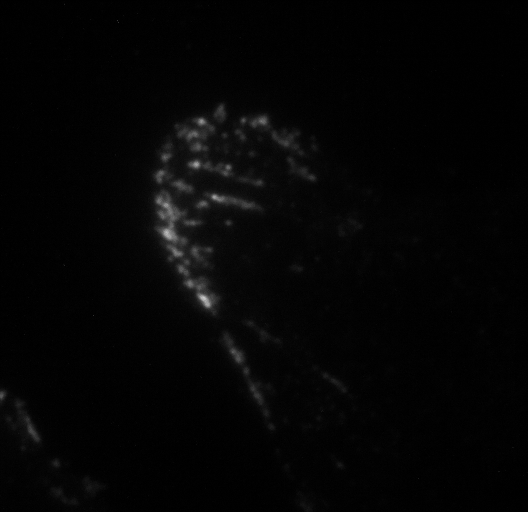

Supplement: S1 Raw data — (ZIP) [file pone.0132174.s002.zip › Final_to_upload/Fig1_paxillin_comp/pax_simD014.tif]

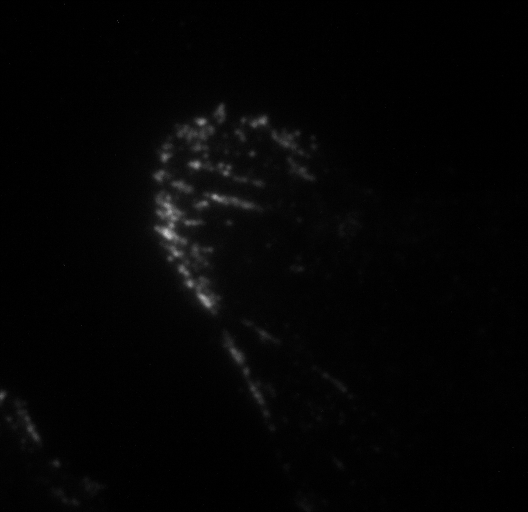

Supplement: S1 Raw data — (ZIP) [file pone.0132174.s002.zip › Final_to_upload/Fig1_paxillin_comp/pax_simD015.tif]

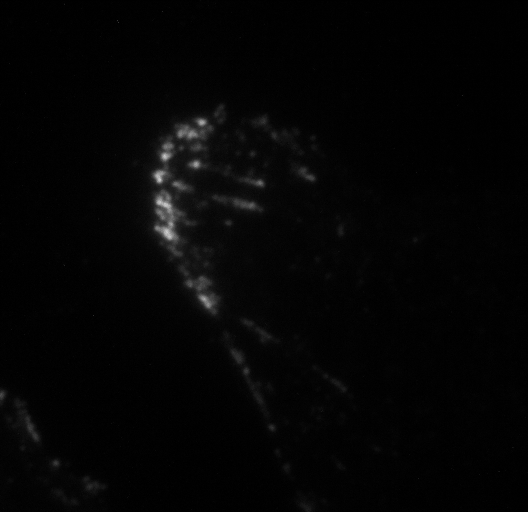

Supplement: S1 Raw data — (ZIP) [file pone.0132174.s002.zip › Final_to_upload/Fig1_paxillin_comp/pax_simD016.tif]

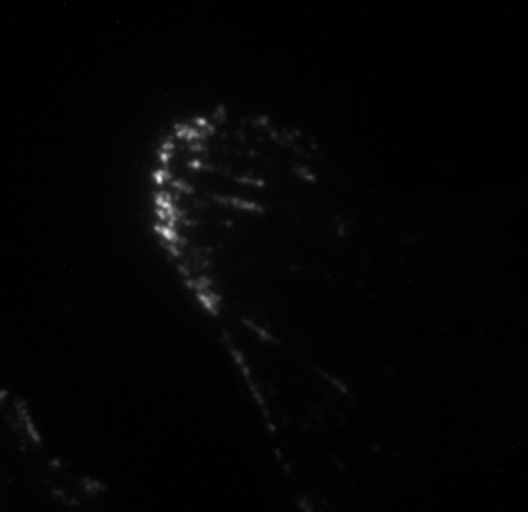

Supplement: S1 Raw data — (ZIP) [file pone.0132174.s002.zip › Final_to_upload/Fig1_paxillin_comp/pax_simD017.tif]

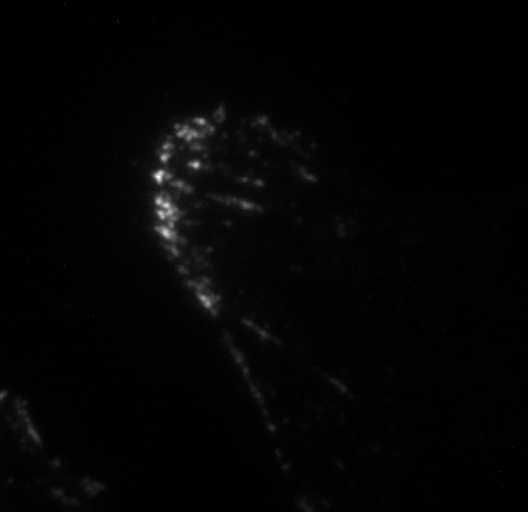

Supplement: S1 Raw data — (ZIP) [file pone.0132174.s002.zip › Final_to_upload/Fig1_paxillin_comp/pax_simD018.tif]

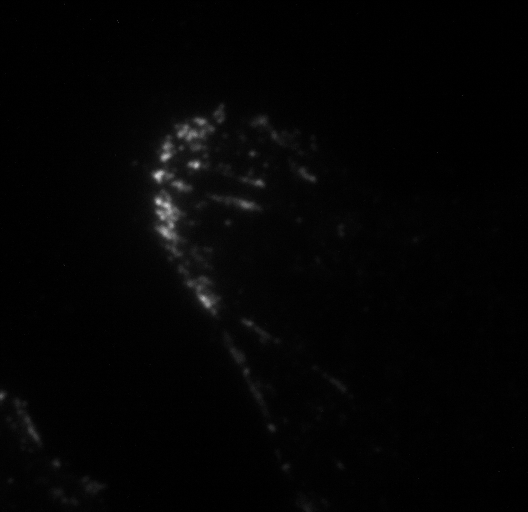

Supplement: S1 Raw data — (ZIP) [file pone.0132174.s002.zip › Final_to_upload/Fig1_paxillin_comp/pax_simD019.tif]

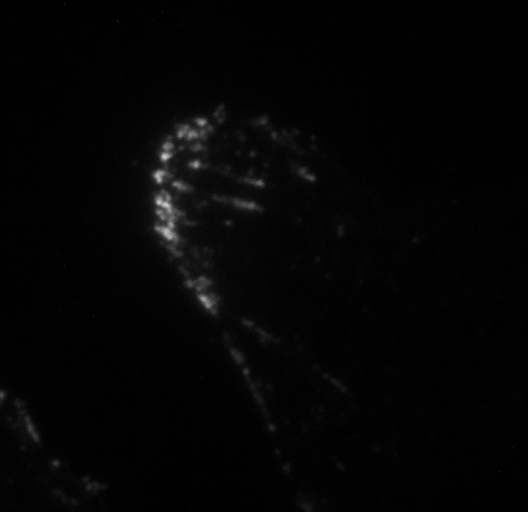

Supplement: S1 Raw data — (ZIP) [file pone.0132174.s002.zip › Final_to_upload/Fig1_paxillin_comp/pax_simD020.tif]

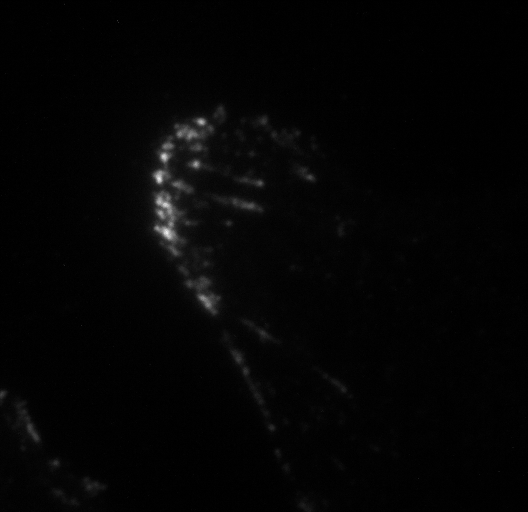

Supplement: S1 Raw data — (ZIP) [file pone.0132174.s002.zip › Final_to_upload/Fig1_paxillin_comp/pax_simD021.tif]

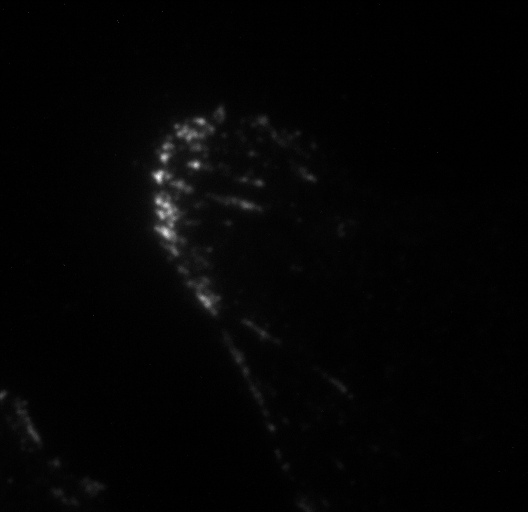

Supplement: S1 Raw data — (ZIP) [file pone.0132174.s002.zip › Final_to_upload/Fig1_paxillin_comp/pax_simD022.tif]

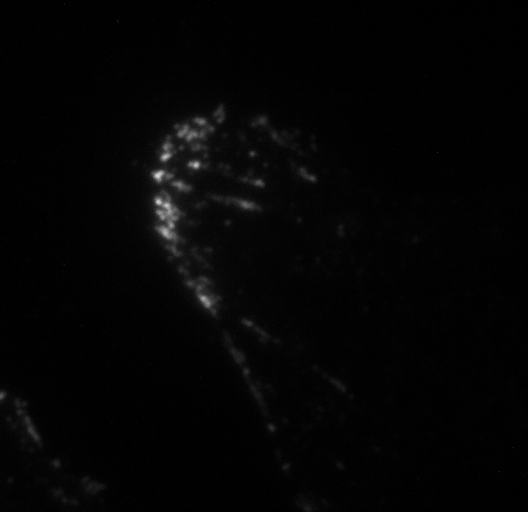

Supplement: S1 Raw data — (ZIP) [file pone.0132174.s002.zip › Final_to_upload/Fig1_paxillin_comp/pax_simD023.tif]

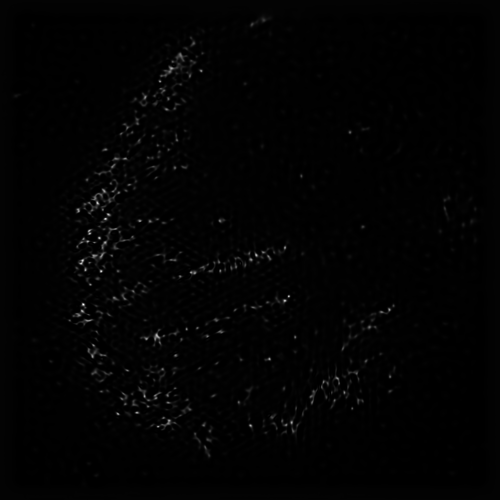

Supplement: S1 Raw data — (ZIP) [file pone.0132174.s002.zip › Final_to_upload/Fig1_paxillin_comp/paxillin_FB_SIM_distortion.tiff]

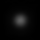

Supplement: S1 Raw data — (ZIP) [file pone.0132174.s002.zip › Final_to_upload/Fig1_paxillin_comp/psf326_corrected.tif]

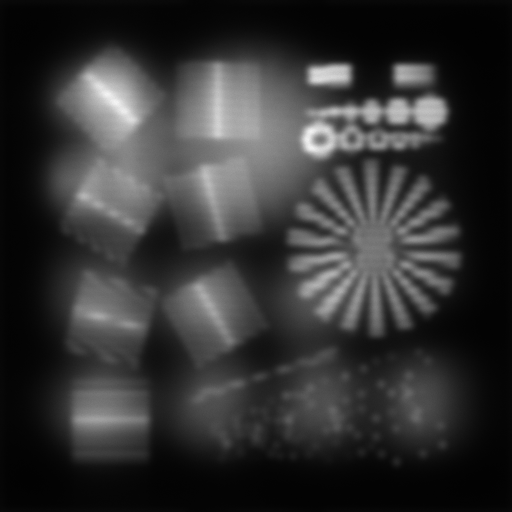

Supplement: S1 Raw data — (ZIP) [file pone.0132174.s002.zip › Final_to_upload/Fig2_simu/raw_simu_data_distorted.tif]

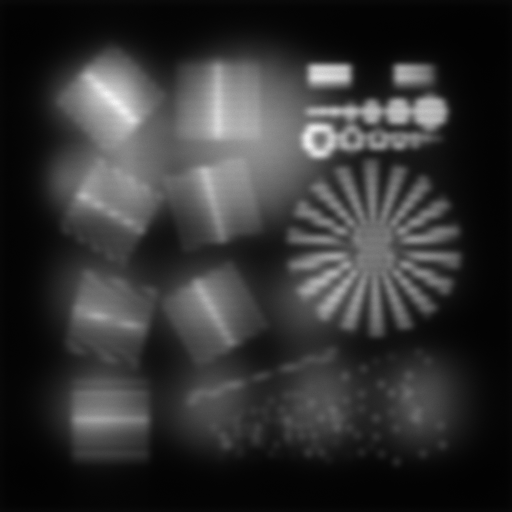

Supplement: S1 Raw data — (ZIP) [file pone.0132174.s002.zip › Final_to_upload/Fig2_simu/raw_simu_data_non_distorted.tif]
